# Forgotten Elastic Band as an Unusual Cause of Limb Ulceration: Case Report and Review of the Literature

**DOI:** 10.1155/2019/6195967

**Published:** 2019-07-14

**Authors:** Keagan Werner-Gibbings, Liesl Ischia, Oleksandr Khoma, Robert Tang

**Affiliations:** Department of Vascular Surgery, Concord Repatriation General Hospital, Hospital Road, Concord, NSW 2139, Australia

## Abstract

We discuss a case of circumferential ulceration of the lower leg in a cognitively impaired elderly man with poor tissue integrity. Thorough clinical examination eventually determined the cause as being a circumferentially placed, forgotten elastic band causing ulceration via sustained tension around the limb. Circumferential application of an elastic band to an extremity is an exceedingly rare but serious cause of lower leg ulceration.

## 1. Introduction

Circumferential application of elastic bands has been previously implicated in the development of significant extremity wounds [1, 2]. Isolated case reports mainly relate to cases of young children [3, 4]. Leg ulceration due to constriction by an inadvertently placed elastic band is far less frequent, especially in the older demographic. We present a case where poor cognition and fragile tissue quality lead to the development and delayed diagnosis of a significant leg wound due to the forgotten application of an elastic band to the lower limb. A forgotten elastic band should be considered as a cause for any circumferential wound of the extremities.

## 2. Case Description

A 79-year-old male presented to a district emergency department having been sent in by his GP for review of a left leg ulcer. His history included type 2 diabetes mellitus. In spite of living independently at home, he was noted to have dementia with significant cognitive impairment.

**Figure 1:**
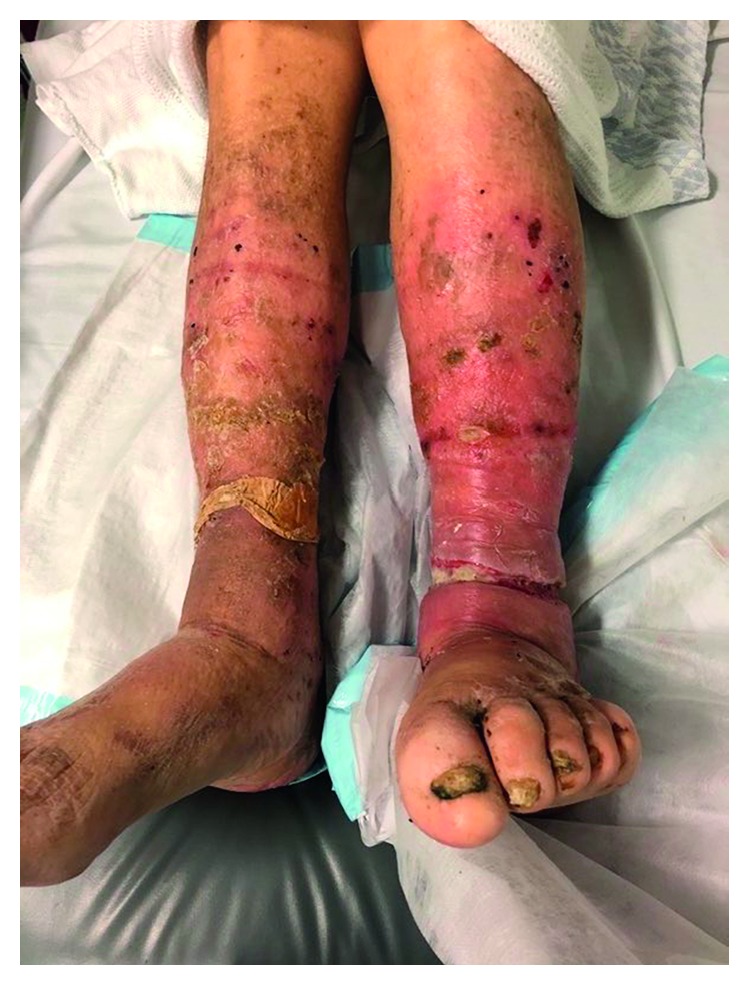
Circumferential leg wound on presentation.

The patient was a vague historian and was unable to give sufficient details regarding the genesis or duration of the wound. He stated the ulcer might have been present for two weeks and had occurred subsequent to a fall. No further information regarding the cause of the ulcer could be elicited. His GP had treated the ulcer unsuccessfully with a one-week course of antibiotics prior to presentation. On examination, the patient was noted to be malnourished and cachectic with significant lower limb oedema. Mini Mental State Examination on review was 12/30. Circumferential ulceration of the left lower leg was noted (Figure 1). The wound was approximately 2 cm wide and extended through the fascia throughout the wound. There was a large burden of tenacious slough, obscuring the base of the wound. The presence of larval infestation was identified. The wound was dressed, antibiotics were commenced, and the patient was transferred to a tertiary referral hospital for review and management by the vascular surgery and geriatric medicine teams.

The patient was reviewed in the emergency department of the tertiary hospital where the wound was inspected before transfer to the ward. A plain radiograph of the area of concern noted a soft tissue defect with no bony abnormality. On review of the wound by the admitting team, the peculiar morphology of the ulcer was noted. The uniform circumferential nature of the wound resembled that of a ligature type injury. Additional questioning however revealed no further clues to elucidate the causative mechanism. The patient denied any process that could adequately explain the circular nature of the lesion. A management plan of elevation, compression, antibiotics, and a dressing regime with the aim of eventual grafting was commenced.

**Figure 2:**
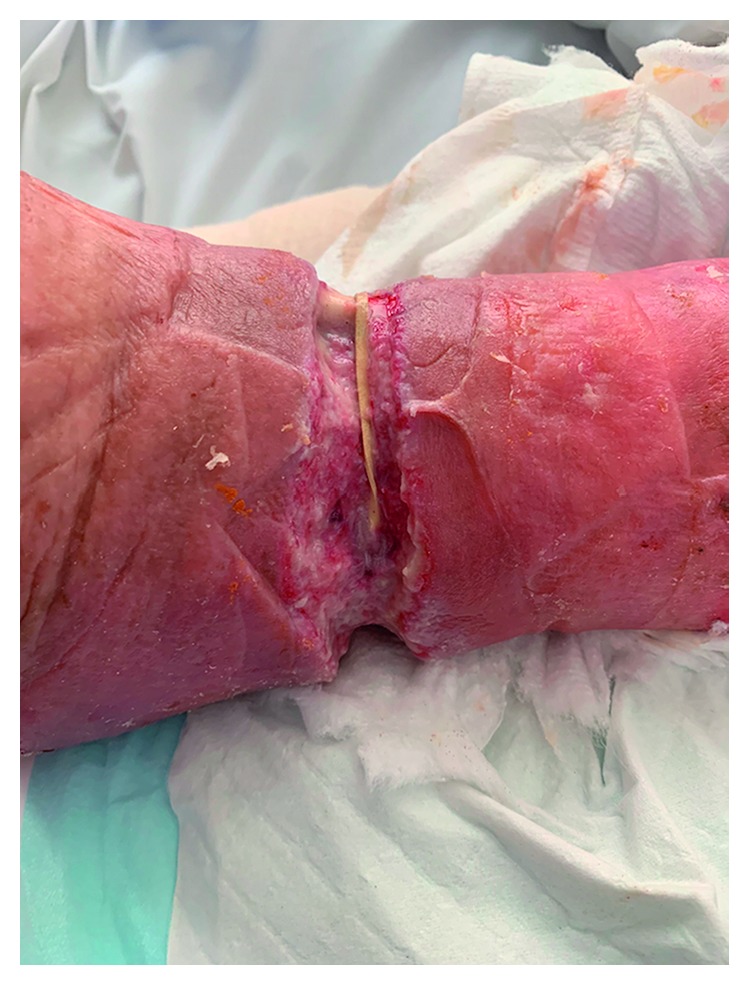
Circumferential leg wound after debridement.

**Figure 3:**
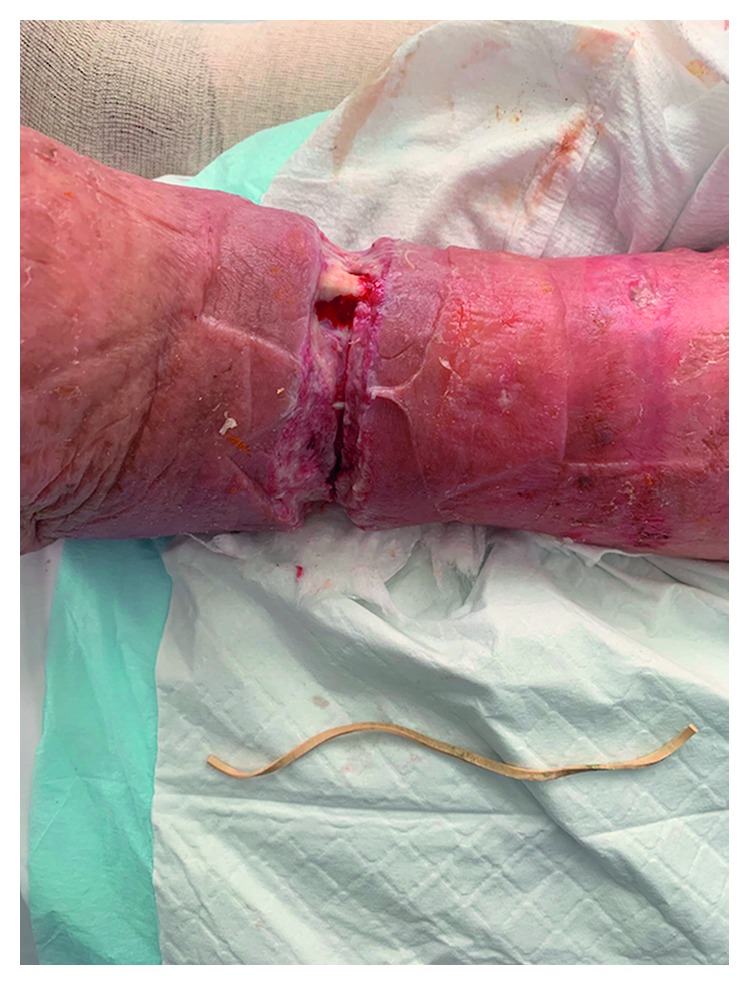
Circumferential leg wound after elastic band removal.

The wound was debrided on the ward, removing the bulk of the tenacious slough covering the wound bed and revealing a clean wound with the base visible. This cleaning demonstrated a thin, tan coloured structure running transversely through the wound. On closer inspection, this was found to be an elastic band, that was constricting the leg down to the level of the fascia and was clearly the aetiological factor precipitating the wound. This had not been previously visible due to the thick layer of slough enveloping the wound. The band was transected and removed (Figures 2 and 3). Further discussion with the patient gave no further insight into how, when, or why the band was placed.

## 3. Discussion

Wounds from circumferential elastic band application are a rare clinical presentation. This unusual presentation highlights two salient points. Firstly, a circumferential elastic band can cause significant tissue injury, especially where tissue quality is poor. Such damage has been previously noted in isolated case reports where the majority of cases relate to young children, with bands placed around the upper limbs causing wounds, infection, and compartment syndrome [5–7]. Leg ulceration is far less frequent, with only one previous case reporting circumferential ulceration in a 77-year-old lady using the band to hold venous dressings in place [2]. While it remains uncertain as to why our patient applied the band, elastic bands can be employed for the purposes of holding garments or dressings in place. As seen here, in those with cognitive issues such as the elderly population, neglecting to remove such bands can cause significant injury.

Of further note was the difficulty with establishing this diagnosis and the extra care that should be taken when unique, unexplained wounds are found. In total, six medical practitioners reviewed the wound before the eventual cause was uncovered, all noting the unusual shape, reminiscent of a ligature wound. While the elastic band was obscured until after the wound was debrided, the unusual and distinctive morphology of the wound should have warranted further investigation. The rarity of this presentation no doubt played a part in the delayed diagnosis; the main factor, however, was the inability of the patient to provide assistance to the medical teams as to the genesis of the wound due to his cognitive impairment. A forgotten elastic band should be considered as a cause for any circumferential wound, especially of the extremities.

## References

[1] Dawson-Butterworth K., Wallen G. D. P., Gittleson N. L. (1969). Self-applied constricting bands. *British Journal of Psychiatry*.

[2] Glew G. (1967). Elastic band injuries. *BMJ*.

[3] Kumar K. M., Shankarappa M. (2013). Rubber band syndrome of the arm. *Journal of Hand and Microsurgery*.

[4] Agarwal A., Kant K. S., Verma I. (2013). The rubber band syndrome: the forgotten rubber band in the wrist. *Hand Surgery*.

[5] John R., Khurana A., Raj N. G., Aggarwal P., Kanojia R., Chayapathi V. (2018). The “forgotten rubber band” syndrome-A systematic review of a uniquely “desi” complication with a case illustration. *Journal of Clinical Orthopaedics and Trauma*.

[6] McIver M. A., Gochman R. F. (2011). Elastic bands on the wrist. *Pediatric Emergency Care*.

[7] Aggarwal A. N., Kini S. G., Arora A., Singh A. P., Gupta S., Gulati D. (2010). Rubber band syndrome–high accuracy of clinical diagnosis. *Journal of Pediatric Orthopaedics*.

